# Post-Sepsis Outcomes: Risk Factors for 90-Day Mortality and Infection Site-Pathogen Similarity in Recurrent Sepsis

**DOI:** 10.4274/TJAR.2026.262525

**Published:** 2026-08-28

**Authors:** Emine Arman Fırat, İlhami Çelik

**Affiliations:** 1 Besni State Hospital, Clinic of Infectious Diseases and Clinical Microbiology, Adıyaman, Türkiye; 2 University of Health Sciences Türkiye, Kayseri City Training and Research Hospital, Clinic of Infectious Diseases and Clinical Microbiology, Kayseri, Türkiye

**Keywords:** Infection site similarity, post-sepsis mortality, post-sepsis syndrome, recurrent sepsis

## Abstract

**Aim:**

Post-sepsis outcomes remain poorly defined, with recurrent infections and late mortality posing significant clinical challenges. To identify clinical and laboratory predictors of 90-day post-sepsis mortality and to evaluate infection site and pathogen similarity in recurrent sepsis.

**Methods:**

This retrospective cohort study included 300 adult patients hospitalized with sepsis or septic shock at a tertiary care center (2018-2022). Demographic, clinical, laboratory, and microbiological data were analyzed. Multivariate bootstrap logistic regression identified independent predictors of mortality, and receiver operating characteristic analysis assessed diagnostic performance. Recurrent episodes were examined for infection site and for microbial concordance with the index hospitalization.

**Results:**

Of 183 discharged patients, 60 (32.8%) died within 90 days. Non-survivors were older, had higher Charlson Comorbidity Index scores, had longer intensive care unit (ICU) stays, had lower albumin and procalcitonin levels, and were less likely to achieve microbiological clearance by day 7. In multivariate analysis, only hypoalbuminemia independently predicted mortality (*P*=0.012). Receiver operating characteristic analysis showed the highest area under the curve for 1/procalcitonin (0.694), followed by age (0.665) and 1/albumin (0.647). Among recurrent sepsis cases, 55.9% had infections at the same site, but identical pathogens were detected in only 5.9%.

**Conclusion:**

Advanced age, comorbidity burden, prolonged ICU stay, delayed microbiological response, and hypoalbuminemia are associated with increased 90-day post-sepsis mortality. Although pathogen concordance was uncommon, a low culture yield indicates that some recurrent episodes may be relapses caused by undetected similar pathogens. When culture-negative episodes are also considered, the true similarity rate between initial and recurrent infections is likely even higher.

**Main Points**

• Ninety-day post-discharge mortality remained high among sepsis survivors, affecting approximately one-third of patients.

• Hypoalbuminemia was the only independent predictor of 90-day mortality in multivariate analysis.

• More than half of recurrent sepsis episodes involved the same anatomical infection site, whereas recurrence with the same pathogen was uncommon.

• Structured post-discharge follow-up may help identify high-risk sepsis survivors and detect recurrent infections at an earlier stage.

## Introduction

Sepsis is a life-threatening systemic inflammatory syndrome that often leads to multiple organ dysfunction as a result of an uncontrolled host response to infection.^1^ While acute in-hospital mortality remains a significant concern, increasing attention has been directed toward post-discharge outcomes, with studies reporting that up to 40% of sepsis survivors are readmitted within the first year-most commonly due to infectious complications.^2^ However, it remains unclear whether these infections represent true recurrences or new infectious events, complicating both diagnosis and clinical management.^3^

The post-sepsis period is frequently marked by persistent immune dysregulation. An initial pro-inflammatory cytokine surge is often followed by a compensatory anti-inflammatory response that includes lymphocyte apoptosis, decreased human leukocyte antigen-DR isotype expression, and regulatory T-cell expansion.^4, 5, 6^ This immunosuppressive state may persist long after hospital discharge, predisposing patients to new infections and increasing late mortality.^4^ Furthermore, recurrent sepsis episodes are more common in sepsis survivors compared to the general population, with rates as high as 35% observed within eight years of the initial episode.^7^

Given these long-term vulnerabilities, it is essential to identify predictors of post-discharge mortality and to characterize the patterns of recurrent sepsis. In this retrospective cohort study, we aimed to investigate the clinical risk factors associated with 90-day mortality following hospital discharge in patients diagnosed with sepsis or septic shock and assess whether recurrent episodes involved infection sites and causative microorganisms similar to those at the initial presentation.

## Methods

### Study Design/Data Source

This single-center, retrospective observational cohort study was conducted at University of Health Sciences Türkiye, Kayseri City Training and Research Hospital. We reviewed records of patients who were hospitalized with sepsis or septic shock between June 1, 2018, and December 31, 2022. Ethics approval for this study was obtained from University of Health Sciences Türkiye, Kayseri City Training and Research Hospital (approval no: 67, date: 24.11.2022). Was conducted in accordance with the principles of scientific ethics.

### Operational Definitions

Patients were analyzed retrospectively over the period from six months prior to admission to 90-days after discharge. Mortality within 90-days post-discharge was recorded. The cause and date of death were obtained from the Kayseri Provincial Health Directorate (petition no: 233655996, date: 08.01.2024). Patients discharged after treatment were categorized into two groups: those who died within 90-days and those who survived.

### Data Collection

Patients with sepsis or septic shock were identified by reviewing discharge summaries, epicrisis notes, and infectious disease consultation notes. Due to potential inaccuracies in diagnostic coding, manual chart reviews were performed to ensure correct case identification.

Sepsis was diagnosed according to the 2016 SCCM/ESICM Sepsis-3 guidelines. Inclusion criteria were age ≥18 years. Exclusion criteria were: pregnancy, concurrent Coronavirus disease-2019 infection, and a history of sepsis within 90-days of the index hospitalization.

Collected data included: Demographic and clinical characteristics: age, gender, comorbidities, total hospital stay, ICU stay, primary infection site, presence of healthcare-associated infections.

• Severity scores: APACHE II, sequential organ failure assessment score (SOFA), glasgow coma scale (GCS), Charlson Comorbidity Index (CCI).

• ICU management parameters: vasopressor use, invasive mechanical ventilation, 7-day microbiological and clinical responses.

Laboratory parameters:

• Hematological: white blood cell count, hemoglobin

• Biochemical: serum creatinine, serum albumin (ALB), alanine aminotransferase, aspartate aminotransferase.

• Inflammatory biomarkers: C-reactive protein, procalcitonin (PCT).

### Statistical Analysis

Statistical analyses were performed using SPSS for Windows, version 22.0 (IBM Corp., Armonk, NY, USA). Descriptive statistics were generated for each group.

• Categorical variables were expressed as frequencies and percentages (%).

• Continuous variables were assessed for normality using the Shapiro-Wilk test and visual inspection of histograms.

• Normally distributed variables were reported as mean ± standard deviation.

• Non-normally distributed variables were expressed as median (interquartile range).

A *P* value ≤0.05 was considered statistically significant.

Univariate analyses were performed to evaluate factors associated with 90-day post-discharge mortality. Variables with *P *< 0.10 in univariate analysis were included in a multivariate model. Multivariate analysis was performed using bootstrap logistic regression with 1,000 resamples to estimate regression coefficients, standard errors, confidence intervals, and *P* values.

The diagnostic performance of selected variables was assessed using receiver operating characteristic (ROC) curve analysis. Variables for ROC analysis were selected based on clinical relevance and statistical significance in univariate or multivariate analyses. The area under the curve (AUC) and 95% confidence intervals were calculated. Optimal cut-off values were determined using the Youden Index, and corresponding sensitivity and specificity values were reported.

For patients with recurrent sepsis, distributions of infection sites and causative microorganisms were compared between episodes, and the similarity of infection sites and microorganisms across recurrent episodes was calculated.

## Results

A total of 300 patients were included in the study. Among them, 183 patients were discharged alive, of whom 60 (32.8%) died within 90-days after discharge, while 123 (67.2%) remained alive during the follow-up period. Baseline demographic, clinical, and laboratory characteristics of patients, stratified by survival status are presented in Table 1. The mean age of the cohort was 75.2±14.2 years, and non-survivors were significantly older than survivors [77.7±12.6 vs. 71.1±16.1 years; crude odds ratio (cOR)=1.032, 95% CI: 1.008-1.058, *P*=0.010]. Gender distribution was similar across groups (*P*=0.792).

The median ICU length of stay was significantly longer in non-survivors than in survivors [6.5 (4-12) vs. 5 (3-11) days; cOR=1.044, 95% CI: 1.006-1.083, *P*=0.024], whereas the total hospital stay did not differ significantly (*P*=0.106). Non-survivors had higher CCI scores than survivors [5 (4-7) vs. 4 (3-6); cOR=1.133, 95% CI: 1.008-1.273, *P*=0.036]. Comorbidities and infection patterns were similar, with a non-significant trend toward more healthcare-associated infections in non-survivors.

Regarding laboratory parameters, non-survivors had significantly lower ALB levels (29.0±4.4 vs. 31.4±5.7 g/L; cOR=0.916, 95% CI: 0.859-0.977, *P*=0.007) and lower median PCT levels [0.3 (0.18-1.37) vs. 3.0 (0.57-27) ng/mL; cOR=0.974, 95% CI: 0.953-0.996, *P*=0.020].

Clinical scores and treatment variables were similar between groups, but the 7-day microbiological response was significantly lower in non-survivors (45.0% vs. 52.0%, *P*=0.044).

In the multivariate bootstrap logistic regression analysis (Table 2), low albumin level was identified as an independent risk factor for 90-day post-sepsis mortality (B=-0.107, 95% CI: -0.243 to -0.029, *P*=0.012). Other variables, including gender, ICU length of stay, CCI, PCT, creatinine, and SOFA score, were not statistically significant predictors after adjustment.

### Diagnostic Performance

**Figure 1:**
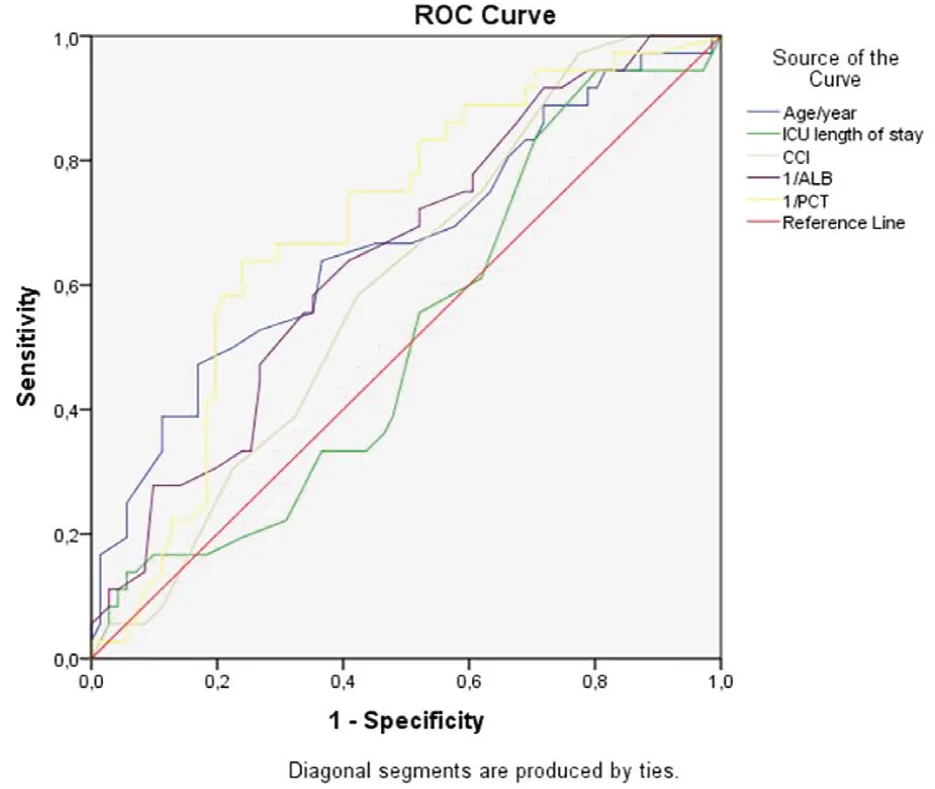
ROC curve. ROC, receiver operating characteristic; CCI, Charlson comorbidity index; ICU, intensive care unit; 1/ALB, 1/serum albumin level; 1/PCT, procalcitonin level.

The ROC analysis results for selected variables are presented in Figure 1 and Table 3. Among the tested variables, 1/PCT had the highest discriminative ability for predicting 90-day mortality (AUC=0.694, 95% CI: 0.591-0.798, *P*=0.001) with a cut-off value of 2.17 (sensitivity: 61.5%, specificity: 77.6%). Age also showed moderate predictive value (AUC=0.665, *P*=0.005, cut-off: 81.5 years), followed by 1/albumin (AUC=0.647, *P*=0.013, cut-off: 0.0293). CCI and ICU length of stay demonstrated low discriminatory performance, with AUC values close to 0.5.

### Similarity Rates of Infection Site and Microorganism Agents of Recurrent Sepsis Cases

Five patients included in the study presented with sepsis on three occasions within 270 days. A total of 68 consecutive episodes of recurrent sepsis were recorded. During the initial hospitalization, respiratory tract infections were most common (36.5%), whereas during the second or third hospitalization, urinary tract infections were most frequent (27%) (Table 4).

In terms of causative microorganisms, culture-negative sepsis was observed in 38% of initial admissions and 29% of recurrent admissions. Gram-negative bacteria were the most common pathogens in both episodes (35% and 38%, respectively), followed by fungi (14% and 12%). No statistically significant differences were observed among pathogen distributions(*P *> 0.05 for all). Among patients with recurrent sepsis, 55.9% had infections at the same anatomical site as during their initial hospitalization, whereas 44.1% had infections at different anatomical sites.

Table 5 presents the similarity rates for both infection sites and causative microorganisms in these recurrent episodes. Among patients with recurrent sepsis, the infection site was similar in 38 patients (60.3%) and different in 30 patients (39.7%). When microbial consistency was compared, similar microorganisms were detected in 5.9% of patients with recurrent infections at the same site and in 2.9% of those with infections at different sites. Notably, 27.9% of recurrent infections at the same site were caused by different microorganisms, indicating that site similarity does not necessarily predict microbiological consistency.

## Discussion

While numerous studies have explored sepsis, there is a paucity of research focusing on mortality in the post-sepsis period. In the literature, patients who were followed for sepsis and discharged following recovery were evaluated, and one-third of them were readmitted to the hospital within the first 90-days after discharge. Sepsis is the most common cause of readmission.^8^ This study investigated post-discharge outcomes among patients who survived sepsis, focusing on 90-day mortality and recurrent sepsis.

Recently, the sepsis survival campaign was established to reduce sepsis-related mortality, and efforts have been made to implement best practices for patient care. The long-term causes of death due to sepsis have been investigated and compared with those of individuals who do not have sepsis; as a result, mortality after sepsis is higher than that in those without sepsis.^9^ The severity of the previous infection, the reaction of the immune system, the cytokine storm that occurs during sepsis, and the reprogramming of cells cause organ dysfunction. Many mechanisms persist after discharge from the hospital and are considered part of post-sepsis syndrome. The most important symptoms observed in post-sepsis syndrome are new or recurrent infections, cardiovascular diseases, changes in protein metabolism, inflammation, immune suppression, fluid accumulation, and cognitive changes. In a review by Gritte et al.^10^ published in 2021, the mortality at 90-days after sepsis was between 27.5-41.3%. In Türkiye, 90-day mortality after sepsis was analyzed in 79 cases and was found to be 31.2%.^11^ In this study, the 90-day post-discharge mortality rate was 32.8%, similar to that reported in other studies.

Many epidemiological studies investigating the causes of post-discharge mortality in patients with sepsis have observed that disease severity increases with age. Age and CCI were reported as important risk factors in a study conducted in France investigating the causes of one-year readmission and mortality of patients after sepsis.^12^ Similarly, in this study, the mean age and CCI of patients who died 90-days after discharge were higher than those of survivors, which is consistent with other studies.

In a study by Gürsoy et al.^11^ examining 90-day mortality after sepsis, the duration of ICU stay was higher in patients who died than in those who survived. In this study, similar to data from Türkiye, ICU length of stay was significantly greater in patients who died after discharge than in patients who ssurvived.

It has long been recognized that serum ALB albumin levels are associated with increased mortality in critically ill patients. Rabi et al.^13^ demonstrated that low albumin levels were strongly associated with higher mortality rates in ICUs, with patients in the low-albumin group showing a general mortality of 71% compared to 52% in the higher-albumin group, and a 28-day mortality of 50% versus 39%. Similarly, Tie et al.^14^ reported that persistently low albumin trajectories in sepsis patients were linked to the poorest outcomes, whereas patients with increasing albumin levels over time had markedly better prognoses. In contrast, stable high albumin levels (>30 g L^-1^) were not associated with significant changes in mortality. Although hypoalbuminemia is linked to worse outcomes, evidence does not yet support routine therapeutic albumin infusion. A 2024 systematic review and meta-analysis concluded that human albumin administration did not significantly reduce overall, ICU, or 90-day mortality in patients with sepsis (pooled RR ~1.0). However, subgroup analysis suggested potential benefit in septic shock when using 20% albumin at higher doses, particularly compared to crystalloids (RR ≈ 0.89-0.90; *P* ≈ 0.03).^15^ In our study, ALB also emerged as an independent predictor of 90-day post-sepsis mortality, which is consistent with recent evidence highlighting its prognostic importance in septic patients.

In our study, median PCT levels were significantly lower in non-survivors than in survivors, and the reciprocal of PCT (1/PCT) demonstrated the highest discriminative ability to predict 90-day post-sepsis mortality (AUC=0.694). In contrast to our findings, the majority of published studies have reported that elevated PCT levels are associated with worse outcomes in sepsis. In particular, persistently elevated or non-declining PCT levels have been associated with increased short-term mortality in patients with sepsis and septic shock.^16^ Meta-analytic evidence indicates that failure to reduce PCT by at least 25% within the first days of therapy is associated with a threefold increase in mortality (pooled RR ~ 3.05; AUC ~ 0.79).^17^ Conversely, some authors have proposed that low PCT concentrations in late or recurrent sepsis may reflect sepsis-induced immunosuppression, a state associated with higher long-term mortality.^18^ Our findings support this hypothesis, suggesting that low PCT in post-sepsis patients may indicate impaired host immune responses rather than low infection severity. Although the univariate analysis in our study supported the latter hypothesis, the association between low PCT and 90-day mortality did not remain statistically significant in multivariate analysis, indicating that the prognostic value of low PCT may be confounded by factors such as comorbidity burden and nutritional status. Therefore, while biologically plausible, low PCT should not yet be considered an independent long-term mortality predictor, and further prospective studies are warranted to clarify its role.

A notable observation was that the seventh-day microbiological response was significantly lower in deceased patients, indicating that persistent or inadequately treated infection may contribute to adverse outcomes. This finding is supported by previous research highlighting the importance of early and effective source control in reducing sepsis-related mortality.^19^ Inadequate microbiological clearance may not only reflect treatment failure but may also predispose patients to recurrent infections and sepsis relapse.

While total hospital length of stay did not differ significantly between groups, ICU stay was longer among patients who died. This may reflect greater initial illness severity, prolonged organ support, or increased risk of nosocomial complications. However, severity scores at admission, such as APACHE II, SOFA, and GCS, were not significantly associated with post-discharge mortality, suggesting that in-hospital physiological instability may not fully capture the long-term vulnerability of sepsis survivors.

It is not always possible to differentiate between infectious and non-infectious causes in patients readmitted after sepsis. Studies on this subject are limited. DeMerle et al.^20^ in a study of patients with sepsis who were followed up and readmitted within 90-days after discharge, the source of infection was similar in 68.6% of 137 patients with recurrent sepsis, the sites of infection were different in 31% and the focus of infection could not be identified in 12%. In a different study conducted by Arshad et al.^21^ in which readmitted patients were examined within 180 days after discharge, recurrent sepsis was detected in 53 patients, with 58% having similar sites of infection and 42% having different sites of infection. Five patients included in this study presented with sepsis or septic shock on three occasions within 270 days, and 68 consecutive episodes of sepsis were recorded. The site of infection was the same in 55.9% of patients, whereas 44.1% had infections at different sites.

In studies examining 90-day post-discharge sepsis-related hospitalizations, the initial hospitalizations and post-discharge readmissions of patients were compared, and similar site infections and similar microorganisms were observed in 19% (1), 22.6%^22^ of patients, respectively, while in a different study examining 180-day post-discharge readmissions, it was shown to be 21%^21^ and in this study it was found to be 5.9%. These findings suggest that recurrent sepsis is not merely a continuation of the initial infection but may represent a new infectious insult in a host with persistent immunologic dysfunction.

### Study Limitations

This study has several limitations. First, it was conducted in a single tertiary care center and had a retrospective design, which may restrict the generalizability of the findings and introduce the risk of missing data or unmeasured confounders. Second, because a high proportion of sepsis cases were culture-negative, pathogen-specific comparisons in recurrent episodes were limited. Third, long-term functional outcomes and quality-of-life measures were not assessed, leaving important aspects of post-sepsis recovery unaddressed. Finally, immunological and inflammatory biomarkers were not evaluated; assessing them could have provided deeper insights into the mechanisms underlying recurrent sepsis and post-sepsis mortality.

## Conclusions

This study demonstrates that advanced age, a higher CCI, a prolonged ICU stay, hypoalbuminemia, unexpectedly low PCT levels, and an inadequate microbiological response are significant predictors of 90-day post-discharge mortality in sepsis survivors. More than half of the patients with recurrent sepsis experienced infections at the same anatomical site. Although microbial concordance was observed in only a small proportion of cases, the low rate of positive cultures raises the possibility that some of these episodes may nevertheless represent relapses caused by similar pathogens that were not detected by microbiological methods. This pattern may reflect both persistent immunosuppression and limitations of culture-based diagnostics in the post-sepsis setting. These findings highlight the need for structured, multidisciplinary post-discharge follow-up, early infection detection, prompt and effective source control, and targeted interventions for high-risk individuals. Further prospective studies integrating advanced microbiological and molecular techniques are warranted to better characterize pathogen recurrence and to develop preventive strategies that reduce both recurrence and long-term mortality.

## Ethics

**Ethics Committee Approval:** Ethics approval for this study was obtained from University of Health Sciences Türkiye, Kayseri City Training and Research Hospital (approval no: 67, date: 24.11.2022).

**Informed Consent:** Because the study was designed retrospectively no written informed consent form was obtained from the patients.

## Figures and Tables

**Table1. Baseline Demographic, Clinical, and Laboratory Characteristics of the Study Population According to 90-Day Post-Discharge Survival Status:** 

-	**Patients included in the study n=300**	**Discharged patients** **n=183**		**cOR (95% CI)**	***P* value**
		**Dead n=60**	**Survivor n=123**		
**Age/year,mean** ± **SD**	75.2±14.2	77.7±12.6	71.1±16.1	1.032 (1.008-1.058)	0.010
**Gender,** **male** **(%)**	154 (51.3)	29 (48.3)	62 (50.4)	1.086 (0.586-2.015)	0.792
**Total** **length** **of** **hospitalization/day,** **median** **(IQR)**	12 (7-19.5)	15 (9-26)	13 (9-18)	1.022 (0.995-1.049)	0.106
**ICU** **length** **of** **stay/day** **median** **(IQR)**	7 (3-13)	6.5 (4-12)	5 (3-11)	1.044 (1.006-1.083)	0.024
**CCI,** **median** **(IQR)**	5 (3-7)	5 (4-7)	4 (3-6)	1.133 (1.008-1.273)	0.036
**Comorbidities** **(%)**					
Hypertension	144 (48)	29 (48.3)	55 (44.7)	1.157 (0.623-2.147)	0.645
Neurological disease	134 (44.7)	32 (53.3)	50 (40.7)	1.669 (0.896-3.107)	0.107
Diabetes mellitus	108 (36)	25 (41.7)	43 (35)	1.329 (0.706-2.503)	0.379
Cardiovascular disease	94 (31.3)	23 (38.3)	33 (36.8)	1.695 (0.880-3.266)	0.115
Chronic renal failure	58 (19.3)	7 (11.7)	21 (17.1)	0.642 (0.256-1.606)	0.343
Chronic lung disease	56 (18.7)	11 (18.3)	24 (19.5)	0.926 (0.420-2.043)	0.849
Malignancy	38 (12.7)	10 (16.7)	12 (9.8)	1.850 (0.750-4.565)	0.182
Pathologies of the urinary system	34 (11.3)	7 (11.7)	18 (14.6)	0.770 (0.303-1.959)	0.584
**Infection** **site** **(%)**					
Urinary tract infection	109 (36.3)	21 (35)	49 (39.8)	0.528 (0.428-1.545)	0.528
Respiratory system	102 (34)	23 (38.3)	33 (26.8)	1.695 (0.880-3.266)	0.115
Skin and soft tissue	39 (13)	7 (11.7)	16 (13)	0.883 (0.343-2.278)	0.797
Gastrointestinal system	22 (7.3)	7 (11.7)	9 (7.3)	1.673 (0.591-4.734)	0.332
Central nervous system	17 (5.7)	-	13 (10.6)	-	-
Multiple regions	11 (3.7)	2 (3.3)	3 (2.4)	1.979 (0.224-8.483)	0.729
**Healthcare-associated** **infection** **(%)**	149 (49.7)	34 (56.7)	57 (46.3)	1.514 (0.813-2.819)	0.191
**Laboratory** **parameters**					
WBC, 10^9^/µL median (IQR)	12.4 (8.9-16.9)	11.9 (8.5-15.5)	12.2 (9-15.3)	1 (1-1)	0.852
HGB, g dL^-1^ mean ± SD	11±2.2	11±2.3	11.9±2	0.887 (0.766-1.027)	0.108
CRE, mg dL^-1^ median (IQR)	1.3 (0.85-2.1)	0.96 (0.79-1.57)	1.2 (0.83-2.2)	0.772 (0.574-1.038)	0.087
ALB, g L^-1^ mean±SD	29±5.6	29±4.4	31.4±5.7	0.916 (0.859-0.977)	0.007
ALT, U L^-1^ median (IQR)	13 (8-26)	13 (9-21)	16 (10-28)	1.002 (0.993-1.011)	0.632
CRP, mg L^-1^ median (IQR)	160 (88-234)	139 (79-238)	149 (80-242)	0.998 (0.995-1.001)	0.208
PCT, ng mL^-1^ median (IQR)	1.5 (0.35-17)	0.3 (0.18-1.37)	3 (0.57-27)	0.974 (0.953-0.996)	0.020
**Clinical** **score** **values** **of** **the** **patients** **at** **admission**					
APACHE II median (IQR)	21 (15-27)	16 (11-24)	17 (13-25)	0.990 (0.950-1.031)	0.617
SOFA median (IQR)	5 (3-8)	3 (2-5)	4 (2-7)	0.887 (0.782-1.007)	0.063
GCS median (IQR)	12 (9-14)	13 (11-14)	13 (11-14)	0.992 (0.873-1.126)	0.898
**Diagnosis** **and** **clinical** **follow-up** **course** **of** **patients** **at** **presentation** **(%)**					
Septic shock	96 (32)	8 (13.3)	19 (15.4)	0.842 (0.346-2.052)	0.705
Administration of vasopressor	136 (45.3)	7 (11.7)	17 (13.8)	0.824 (0.322-2.108)	0.686
Invasive mechanical ventilation	113 (37.7)	1 (1.7)	1 (0.8)	-	-
Seventh-day clinical response	163 (54.3)	49 (81.7)	100 (81.3)	1.025 (0.462-2.270)	0.952
Seventh-day microbiological response	126 (42)	27 (45)	64 (52)	0.328 (0.111-0.971)	0.044

ALB, serum albumin; ALT, alanine aminotransferase, APACHE II, Acute Physiology and Chronic Health Evaluation II; CCI, Charlson comorbidity index; CRE, serum creatinine; CRP, C-reactive protein; cOR, crude odds ratio (95% confidence interval); GCS, Glasgow Coma scale; HGB, hemoglobin; ICU, intensive care unit; IQR, interquartile range; PCT, procalcitonin; SD, standard deviation; SOFA, sequential organ failure assessment score; WBC, white blood cell count

**Table 2. Bootstrap Logistic Regression Analysis and Odds Ratios for 90-Day Post-Sepsis Mortality:** 

**Variable**	**B**	**Bootstrap SE**	**Bootstrap *P* value**	**95% CI lower**	**95% CI upper**
Gender, male	0.394	0.603	0.458	-0.748	1.617
ICU, length of stay	0.012	0.037	0.653	-0.076	0.073
CCI	0.084	0.098	0.303	-0.066	0.328
ALB	-0.107	0.054	**0.012**	-0.243	-0.029
PCT	-0.025	0.152	0.108	-0.43	-0.003
CRE	-0.127	0.309	0.569	-0.911	0.354
SOFA	0.012	0.121	0.901	-0.229	0.234
Constant	2.232	1.909	0.157	-0.969	6.829

ALB, serum albumin; CCI, Charlson comorbidity index; CRE, serum creatinine; ICU, intensive care unit; PCT, procalcitonin; SOFA, sequential organ failure assessment score

**Table 3. ROC Analysis Results: Diagnostic Performance of Clinical Variables in Predicting Mortality:** 

**Variable**	**AUC**	**Std. error**	***P* value**	**95% lower**	**95% CI upper**	**Cut-off**	**Sensitivity (%)**	**Specificity (%)**
Age	0.665	0.057	0.005	0.553	0.778	81.5	50	74.9
ICU length of stay	0.516	0.058	0.784	0.402	0.63	3.5	87.5	34.5
CCI	0.599	0.055	0.095	0.491	0.707	2.5	96.7	20.3
1/ALB	0.647	0.055	0.013	0.539	0.755	0.0293	93.1	28.6
1/PCT	0.694	0.053	0.001	0.591	0.798	2.17	61.5	77.6

CCI, Charlson comorbidity index; ICU, intensive care unit; 1/ALB, 1/serum albumin level; 1/PCT, procalcitonin level

**Table 4. Comparison of Infection Sites and Causative Microorganisms in Recurrent Sepsis Patients:** 

***-***	**Initial hospitalization of patients with recurrent sepsis**	**Second or third hospitalization of patients with recurrent sepsis**	***P***
Number of patients, n	**n=63**	**n=68**	***-***
**Infection** **sites** **(%)**			
Respiratory system	23 (36)	23 (34)	0.748
Urinary tract infection	22 (35)	27 (40)	0.572
Gastrointestinal system	5 (8)	3 (4)	0.400
Skin and soft tissue	10 (16)	11 (16)	0.962
Central nervous system	1 (2)	2 (3)	-
Multiple regions	2 (3)	2 (3)	-
**Causative** **microorganisms** **in** **recurrent** **sepsis** **patients** **(%)**			
Culture is negative	24 (38)	20 (29)	0.293
Gram-negative bacteria	22 (35)	26 (38)	0.694
Gram-positive bacteria	3 (5)	8 (12)	0.149
Polymicrobial	5 (8)	6 (9)	0.855
Fungus	9 (14)	8 (12)	0.668

**Table 5. Similarity Rates of Infection Site and Causative Microorganism in Recurrent Sepsis Patients:** 

**Microorganism reproduction**	**Infection site**	
	**Similar site infection (%)**	**Infection of different regions (%)**
	**n=38**	**n=30**
Culture negative/reproduction	6 (8.8)	9 (13.2)
Reproduction/culture negative	4 (5.9)	8 (11.8)
Culture negative/culture negative	5 (7.3)	4 (5.9)
**Reproduction/reproduction**	23 (33.8)	9 (13.2)
Similar microorganism	4 (5.9)	2 (2.9)
Different microorganism	19 (27.9)	7 (10.3)
